# Calreticulin: Roles in Cell-Surface Protein Expression

**DOI:** 10.3390/membranes4030630

**Published:** 2014-09-16

**Authors:** Yue Jiang, Sandeepa Dey, Hiroaki Matsunami

**Affiliations:** 1 Department of Molecular Genetics and Microbiology, University Program of Genetics and Genomics, Duke University Medical Center, Durham, NC 27710, USA; 2 Department of Cell Biology, The Scripps Research Institute, 10550 North Torrey Pines Road, La Jolla, CA 92037, USA; E-Mail: deys@scripps.edu; 3 Department of Neurobiology, Duke Institute for Brain Sciences, Duke University Medical Center, Durham, NC 27710, USA

**Keywords:** calreticulin, calreticulin4, calnexin, endoplasmic reticulum, chaperone, trafficking, receptor, channel, retention, vomeronasal, V2R, CFTR

## Abstract

In order to perform their designated functions, proteins require precise subcellular localizations. For cell-surface proteins, such as receptors and channels, they are able to transduce signals only when properly targeted to the cell membrane. Calreticulin is a multi-functional chaperone protein involved in protein folding, maturation, and trafficking. However, evidence has been accumulating that calreticulin can also negatively regulate the surface expression of certain receptors and channels. In these instances, depletion of calreticulin enhances cell-surface expression and function. In this review, we discuss the role of calreticulin with a focus on its negative effects on the expression of cell-surface proteins.

## 1. Introduction

Since its first identification in rabbit skeletal muscles [1], calreticulin has been found in many organisms from plants to animals. Calreticulin is primarily localized in the endoplasmic reticulum (ER), where it functions as a chaperone. Together with its membrane-anchored homolog calnexin, calreticulin assists the folding and controls the quality of newly synthesized glycoproteins that enter the ER before they are exported to various subcellular compartments [2]. Proteins that are eventually sorted to the cell surface, including receptors and channels, are an important group of substrates for calreticulin. However, a few lines of evidence argue that the quality control by ER-resident calreticulin can sometimes be over-stringent, retaining “non-native” proteins with mutations or of heterologous origins inside the ER, even though these proteins would otherwise be functional at the cell surface. Moreover, in addition to the ER, calreticulin is found at multiple subcellular localizations, where it mediates a variety of cellular processes including apoptotic cell clearance, cell adhesion, and cell migration [3,4,5]. Calreticulin in post-ER compartments can also down-regulate the expression of certain molecules at the cell surface, presumably by promoting their internalization and degradation [6]. In these instances, depletion of calreticulin facilitates the surface expression of certain receptors and channels, thus enhancing their functions. The negative effects of calreticulin on cell-surface protein expression are of particular interest for at least two reasons. First, many human genetic disorders are caused by mutations that lead to ER retention of the encoded proteins and affect their trafficking to the cell surface [7]. ER retention can directly contribute to diseases, when mutations themselves do not severely affect protein function. Understanding the role of calreticulin in these circumstances may facilitate the treatment of these diseases. Secondly, the functional characterization of cell surface proteins, such as receptors and channels, largely relies on heterologous expression systems. However, heterologously expressed cell-surface proteins trapped by calreticulin can compromise investigation of these proteins. In this review, we first introduce the known functions of calreticulin in and outside the ER, and then provide examples to discuss the undesired effects of calreticulin on protein surface expression. 

## 2. Calreticulin in the ER

**Figure 1:**
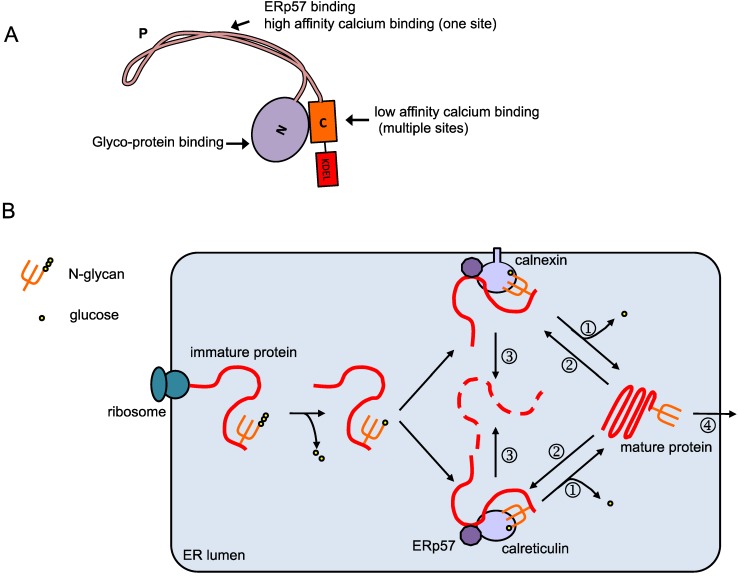
Overview of the calreticulin protein and its function. (**A**) Domain organization of calreticulin. The calreticulin protein contains the N-terminal signal peptide, N-domain, P-domain, C-domain and the ER-retention signal KDEL. The N- and P- domain are responsible for the chaperone function, while the C-domain is mainly responsible for calcium binding; (**B**) Calreticulin/calnexin cycle. Native protein peptides being synthesized on the rough ER translocate into the ER lumen and are glycosylated. The glycosylated protein is then modified by glucosidases I and II, and binds to calreticulin and/or calnexin. The oxidoreductase ERp57 associates with calreticulin and calnexin, and catalyzes the formation and breakage of protein disulfide bonds to assist folding. When folding is completed, glucosidases II further trims the glycan (①) and the glycoprotein is released from calreticulin/calnexin and transported out of the ER (④). Incompletely-folded proteins are re-glucosylated by UGGT (UDP-glucose:glycoprotein transferase, ②), and remain bound to calreticulin/calnexin to continue folding. Prolonged interaction with calreticulin/calnexin targets the proteins to ERAD (ER-associated degradation, ③).

In 1974, Ostwald *et al*. isolated a calcium-binding protein from the ER of rabbit skeletal muscles [1]. 15 years later, the gene coding for this protein was cloned and the product was named “calreticulin”, reflecting its ability to bind calcium and its primary localization to the endoplasmic reticulum [8,9]. The sequence of calreticulin suggests a zonal organization of this protein: a neutral N-terminal region (N-domain), a proline-rich region with internal repeats (P-domain), and an acidic C-terminal region (C-domain) that terminates with the KDEL (Lys-Asp-Glu-Leu) ER retention signal (Figure 1A). The N-domain contains a high affinity calcium-binding site [10], while the C-domain contains multiple low affinity calcium binding sites that are involved in regulating calcium homeostasis [11]. Shortly after its cloning, the chaperone function of calreticulin was proposed based on the significant homology of the P-domain with calnexin [12], which was identified as a membrane-integrated ER chaperone facilitating biogenesis of major histocompatibility complex (MHC) class I molecules [13,14,15]. Moreover, calreticulin is also involved in the assembly of MHC class I molecules [16]. Today, we know how calreticulin, together with calnexin, forms the calreticulin/calnexin cycle that assists folding of glycoproteins that traverse through the ER [2,17,18,19] (Figure 1B). Native proteins being synthesized in ribosomes on the rough ER translocate into the ER lumen. Upon entering the ER, a pre-assembled oligosaccharide (Glc_3_ Man_9_ GlcNAc_2_) is added to the asparagine (N) residue of the protein, by the recognition of a specific sequence on the newly synthesized protein. The N-glycosylated protein is then modified by glucosidases I and II, which, respectively, remove one and two glucose residues from the N-linked glycan. Before glucosidase II modifies the glycan further, the glycosylated protein is recognized by calreticulin and/or calnexin. During the time span in which the substrate protein remains bound to these chaperones, the protein reaches its mature conformation, and is eventually released and transported out of the ER after glucosidase II removes more glucose residues. Incompletely-folded proteins are recognized and re-glucosylated by UDP-glucose:glycoprotein transferase (UGGT), and are retained in the calreticulin/calnexin cycle until properly folded. ERp57 is an oxidoreductase physically associated with calreticulin and calnexin, which catalyzes the formation and breakage of disulfide bonds to assist protein folding [20,21]. Proteins with prolonged interaction with calreticulin/calnexin are identified as “misfolded” and directed to the ER-associated degradation (ERAD) pathway. Thus, together with calnexin and other chaperones, calreticulin effectively marks misfolded proteins and ensures only correctly folded proteins exit the ER. As an ER chaperone, calreticulin is important for the normal trafficking of many cell surface proteins. Mouse embryonic fibroblasts deficient in calreticulin exhibited increased ER retention of collagen, the extracellular matrix component [22]. Calreticulin up-regulates the expression of epithelial sodium channel (ENaC) subunits, and increased channel activities were observed in cells overexpressing calreticulin [23]. Calreticulin has also been shown to stabilize and promote the stability of human insulin receptors in the ER [24]. Cell surface expression of sterol transporters, specifically ATP-binding cassette (ABC) G5/G8, is also facilitated by calreticulin [25].

## 3. Calreticulin Outside the ER

Early studies showed that calreticulin was found on the surface of many mammalian cells [26,27,28]. One known function of cell surface calreticulin is its involvement in focal adhesion disassembly. Thrombospondin (TSP) interacts with cell surface calreticulin and signals through low density lipoprotein receptor-related protein (LRP) to promote focal adhesion disassembly and cell migration [29,30]. Perhaps as a reflection of these functions, topically applied calreticulin increased the rate of wound healing [4]. Another important function of calreticulin at the cell surface is to mediate phagocytosis of apoptotic or dying tumor cells. Calreticulin was first found to function on phagocytic cells as a receptor for C1q [31,32,33,34,35]. Later on, calreticulin was also shown to serve as an “eat me” signal on the surface of apoptotic cells, which is recognized by LRP on phagocytic cells [3]. Interestingly, calreticulin surface exposure is not simply a result of passive exposure of ER contents during cell death, but a preapoptotic event that appears highly regulated. For example, anthracycline used in cancer chemotherapy induces calreticulin exposure in cancer cells within one hour, preceding the apoptosis-associated phosphatidylserine exposure. This calreticulin translocation is important for the immunogenicity of dying tumor cells, which is key to the success of chemotherapy [36]. Elements of the ER stress response, including the activation of protein kinase RNA-like ER kinase (PERK) and subsequent phosphorylation of eIF2α, are required for calreticulin exposure during immunogenic cell death [37]. In addition, reduction of calcium levels in the ER also favors the surface exposure of calreticulin [38]. Calreticulin exposure also requires the apoptotic pathway, including activation of caspase-8, subsequent cleavage of the ER protein BAP31 and activation of BAX and BAK [37]. The translocation of calreticulin to the cell surface requires the anterograde transport system from the ER to the Golgi, and the SNARE-dependent exocytosis pathway [37]. 

## 4. Calreticulin as an ER-Retention Factor

As an ER-resident chaperone, calreticulin keeps control of both exogenous and endogenous proteins. Molinari *et al*. examined the impact of calreticulin on the heterologous expression of several well-studied viral proteins, including influenza virus hemagglutinin (HA), as well as Semliki forest virus (SFV) E1 and p62 [39]. They found that in mouse embryonic cells deficient for calreticulin, these viral proteins were able to mature, and traffic to the cell surface normally, suggesting that calreticulin is not strictly required for the folding of these proteins. Interestingly, pulse-chase experiment showed that the maturation of these viral proteins were in fact accelerated in the absence of calreticulin, indicating that calreticulin might be an ER-retention factor for heterologously expressed proteins. In addition, calreticulin is also an important ER-retention factor for mutated cell surface proteins, such as truncated tyrosinase that causes oculocutaneous albinism [40]. 

ER retention of exogenous proteins is sometimes undesired, especially for attempts to express transmembrane or secreted proteins for functional analyses. For instance, many chemosensory receptors are difficult to express in heterologous cells, including the mammalian vomeronasal receptors, the V2Rs [41,42,43]. V2Rs are a class of putative pheromone receptors that are expressed in the vomeronasal organ (VNO). These receptors detect chemosignals and mediate innate social behaviors, such as mating and aggression. When expressed in heterologous cells, such as HEK293T, V2Rs are retained in the ER and fail to traffic to the cell surface, largely impeding the functional analysis of these receptors. It has been shown that depleting calreticulin in HEK293T cells allows V2Rs to be expressed at the cell surface [44], suggesting calreticulin is a retention factor for V2Rs. In a calreticulin-knockdown HEK293T cell line, with the addition of a few co-factors, two V2Rs were functionally expressed at the cell surface and showed response to peptide ligands. Interestingly, *in situ* hybridization showed that the VNO highly expresses a calreticulin homolog, calreticulin4 (Calr4), instead of calreticulin. The down regulation of calreticulin in the VNO was also confirmed at protein level. While calreticulin and Calr4 are about 67% identical, the N, P and C domains can be broadly identified in Calr4 as well. Certain residues in calreticulin that have been shown to be critically important for its function (E238, D241, W244, W302) as a protein-folding chaperone [45] are also conserved in Calr4. Indeed, Calr4 at least partially substitutes for calreticulin as a chaperone, as it alleviated the ER stress and unfolded protein responses caused by calreticulin depletion in HEK293T cells. However, while calreticulin stably interacts with V2Rs and retains them in the ER, Calr4 does not. While the molecular basis of the different affinities for V2Rs between Calr4 and calreticulin is yet to be determined, it appears that the VNO expresses a specific type of calreticulin, Calr4, that is compatible with functional expression of V2Rs. Consistent with this idea, analysis of draft or assembled genomes of several vertebrates showed pseudogenizing mutations in Calr4, particularly in species that did not have a functional V2R repertoire. 

Similarly, although retention of mutated cell surface protein is generally a protective mechanism, in certain cases the mutated protein is in fact functional. In these cases, the retention itself can be the cause of defects. In addition to the V2R example in mammals, calreticulin-specific retention of cell surface receptors is also reported in plants. BRI1 is a cell surface receptor for brassinosteroids (BRs), a class of plant steroid hormones that is crucial for the plant growth [46]. A point mutation in BRI1 (*bri1-9*) results in ER retention of this receptor, and leads to a dwarf phenotype in *Arabidopsis thaliana* [47]. Li and colleagues performed an EMS mutagenesis screen for suppressor of the dwarf phenotype in *bri1-9* plants, and recovered *ebs* (*EMS-mutagenized bri1 suppressor*) mutants [48]. These *ebs* mutants are allele-specific for *bri1-9* that retains the point mutated receptor in the ER, *i.e*., they cannot rescue other BRI1 mutant alleles such as BRI1 deletion, suggesting that these *ebs* mutants do not activate BR signaling in parallel to BRI1. Instead, these *ebs* mutants were mapped to genes involved in ER quality control, with *ebs2* mapping to a calreticulin gene CRT3. Specifically, in *ebs2* mutants deficient for CRT3, BRI1 is released from the ER in spite of the point mutation on the receptor. As the receptor reaches the cell surface it largely restores the BR signaling. Many plants have multiple calreticulins that belong to two classes: CRT1/2 and CRT3. Only the CRT3 mutant, but not mutants of CRT1, CRT2 or calnexin, rescues the BR signaling defect of *bri1-9*. In line with this, only CRT3 immunoprecipitates with *bri1-9*. The C-domain is the most divergent region between CRT1/2 and CRT3, and domain-swapping experiments show that the C-domain of CRT3 is crucial for retaining the mutant *bri1-9* in the ER. The authors speculated that the C-domain might assign unique sub-organelle localization pattern for CRT3 to facilitate its interaction with *bri1-*9. Thus, in this case, a mutant cell-surface receptor in plant is retained in the ER, and can be released by the deficiency of one specific calreticulin but not other calreticulins. It is important to note that the receptor, although containing a mutation, remains largely capable of its designated function, as it rescues the dwarf phenotype when allowed to traffic to the cell surface. Interestingly, mapping of other *ebs* mutant strains recovered mutations in other components of the calreticulin/calnexin cycle, including glycosyltransferases ALG9 and ALG12 which are responsible for the assembly of the glycan added to the newly synthesized protein when it translocates into the ER to enter the calreticulin/calnexin cycle [49,50], UGGT that transfers the glucose residue back to the substrates to promote their association with calreticulin/calnexin [47], and Hrd1, an ER membrane-localized ubiquitin ligase that directs proteins retained in calreticulin/calnexin cycle to ERAD [51]. 

In summation, the above examples demonstrate how over-stringent ER quality control systems can affect protein surface expression. Specific calreticulins can display substrate-specific affinities, by which a certain calreticulin but not others becomes the retention factor that prevents the trafficking of certain proteins to the cell surface.

## 5. Calreticulin at the Cell Surface Can Destabilize Cell-Surface Protein

Although primarily located in the ER, calreticulin can be found outside the ER to function in diversified processes *in situ*. Accordingly, it has also been reported that calreticulin at the post-ER compartments inhibits the cell surface expression of proteins, as in expression of the cystic fibrosis transmembrane conductance regulator (CFTR), a cell surface cAMP-dependent Cl^−^ channel [6]. Mutations of this channel result in cystic fibrosis, the most common lethal genetic disease in Caucasians. The most common mutation of CFTR, CFTR ΔF508, fails to mature and is retained in the ER [52]. Forced expression of calreticulin is shown to down-regulate wild type CFTR expression and function at the cell surface in cell culture system. Accordingly, RNAi-mediated knockdown of calreticulin enhances the function of CFTR. However, depletion of calreticulin does not facilitate the maturation of CFTR, as the mutant CFTR ΔF508 is still trapped in the ER and fails to mature in calreticulin deficient cells. In contrast, calreticulin regulates the surface expression of CFTRs at post-ER compartments. Pulse-chase experiments show that the overexpression of calreticulin enhances the internalization and proteasome degradation of mature CFTR at the cell surface. In line with this, calreticulin deletion mutants lacking the entire C terminus or the KDEL ER retention signal, both of which exit the ER, are even more potent than wild type calreticulin in terms of inhibiting CFTR surface expression. Although calreticulin deficiency alone does not correct the function of CFTR ΔF508, when combined with the permeable temperature (25 °C) that allows some mutant CFTR to reach the cell surface, calreticulin deficiency stabilizes CFTR ΔF508, thus enhancing the function of the channel. Interestingly, curcumin, which is shown to correct cystic fibrosis caused by CFTR ΔF508, appears to function by down-regulating the expression of calreticulin [53]. At the permeable temperature, administration of curcumin shows similar effects as calreticulin depletion in enhancing the mutant CFTR function. Thus, calreticulin can decrease the stability of cell-surface proteins. In addition, it is possible to target calreticulin with drugs, thus enhancing the stability and function of mutated cell-surface proteins involved in human diseases.

Overexpression of calreticulin has also been shown to decrease the surface expression of neuroendocrine Cav1.3 L-type calcium channels in human fetal cardiomyocytes [54]. Interestingly, calreticulin was also found at the cell surface in this study, however it is not clear whether the cell surface calreticulin is directly responsible for the reduction in Cav1.3 surface expression levels.

## 6. Outlook

**Figure 2:**
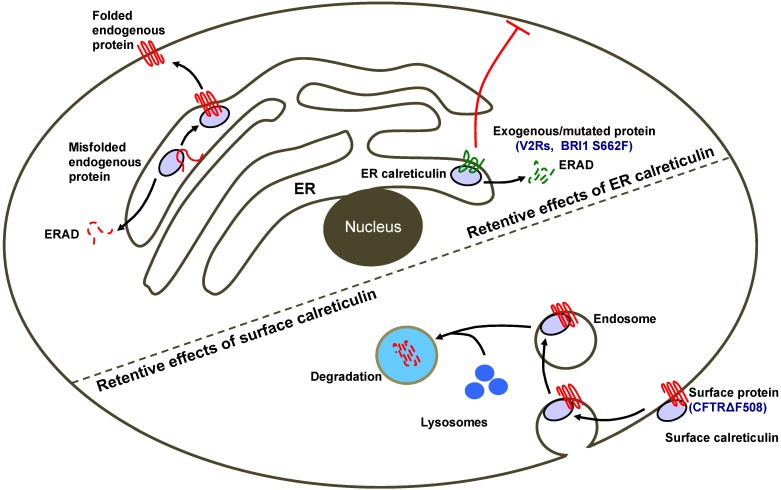
Mechanisms for calreticulin-mediated inhibition of cell surface protein localization. Calreticulin in the ER can trap misfolded endogenous proteins and exogenous/mutated proteins, preventing them to be exported to the cell surface and target them to ERAD (ER-associated degradation). In addition, calreticulin in post-ER compartments can facilitate the internalization of proteins into endosomes, inhibiting their recycling and promoting degradation, thus removing them from the cell surface [6].

Calreticulin can inhibit the surface expression of a diverse set of proteins before and after they are trafficked out of the ER (Figure 2), but many aspects of the underlying mechanism remain unclear. First, not all cell surface proteins are down regulated by calreticulin. Thus, although inhibiting calreticulin promotes the surface expression of the proteins of interest in several cases, the generality of this approach is yet to be determined. Second, more work is required to understand how calreticulin retains certain proteins in the ER, and how the cargo specificities are achieved. Calreticulin and calnexin, and possibly different calreticulins, may have overlapping but non-identical substrates. While the current model involves direct binding of calreticulin to the cargo for ER retention, it is also possible that the calreticulin effect is indirect; calreticulin might prevent the interaction between the cargo and other chaperones to retain the cargo in the ER. Third, the mechanism of how cell surface calreticulin regulates protein stability is unclear. Calreticulin enhances the internalization and proteasome degradation of mature CFTR, but the underlying mechanism is missing. One possibility is that cell surface calreticulin and CFTR are co-internalized, since calreticulin is shown to interact with CFTR at the cell surface. In spite of the unanswered questions, the negative regulation of cell surface proteins by calreticulin is worth noting, especially when surface expression is desired. In such circumstances, inhibiting calreticulin with small molecules or depleting calreticulin expression by siRNAs or other technologies may improve the surface expression of the proteins of interest.

## References

[1] Ostwald T.J., MacLennan D.H. (1974). Isolation of a high affinity calcium-binding protein from sarcoplasmic reticulum. J. Biol. Chem..

[2] Michalak M., Groenendyk J., Szabo E., Gold L.I., Opas M. (2009). Calreticulin, a multi-process calcium-buffering chaperone of the endoplasmic reticulum. Biochem. J..

[3] Gardai S.J., McPhillips K.A., Frasch S.C., Janssen W.J., Starefeldt A., Murphy-Ullrich J.E., Bratton D.L., Oldenborg P.A., Michalak M., Henson P.M. (2005). Cell-surface calreticulin initiates clearance of viable or apoptotic cells through trans-activation of lrp on the phagocyte. Cell.

[4] Gold L.I., Eggleton P., Sweetwyne M.T., Van Duyn L.B., Greives M.R., Naylor S.M., Michalak M., Murphy-Ullrich J.E. (2010). Calreticulin: Non-endoplasmic reticulum functions in physiology and disease. FASEB J. Off. Publ. Fed. Am. Soc. Exp. Biol..

[5] Martins I., Kepp O., Galluzzi L., Senovilla L., Schlemmer F., Adjemian S., Menger L., Michaud M., Zitvogel L., Kroemer G. (2010). Surface-exposed calreticulin in the interaction between dying cells and phagocytes. Ann. N.Y. Acad. Sci..

[6] Harada K., Okiyoneda T., Hashimoto Y., Ueno K., Nakamura K., Yamahira K., Sugahara T., Shuto T., Wada I., Suico M.A. (2006). Calreticulin negatively regulates the cell surface expression of cystic fibrosis transmembrane conductance regulator. J. Biol. Chem..

[7] Brooks D.A. (1997). Protein processing: A role in the pathophysiology of genetic disease. FEBS Lett..

[8] Smith M.J., Koch G.L. (1989). Multiple zones in the sequence of calreticulin (crp55, calregulin, hacbp), a major calcium binding er/sr protein. EMBO J..

[9] Fliegel L., Burns K., MacLennan D.H., Reithmeier R.A., Michalak M. (1989). Molecular cloning of the high affinity calcium-binding protein (calreticulin) of skeletal muscle sarcoplasmic reticulum. J. Biol. Chem..

[10] Kozlov G., Pocanschi C.L., Rosenauer A., Bastos-Aristizabal S., Gorelik A., Williams D.B., Gehring K. (2010). Structural basis of carbohydrate recognition by calreticulin. J. Biol. Chem..

[11] Mesaeli N., Nakamura K., Zvaritch E., Dickie P., Dziak E., Krause K.H., Opas M., MacLennan D.H., Michalak M. (1999). Calreticulin is essential for cardiac development. J. Cell Biol..

[12] Krause K.H., Michalak M. (1997). Calreticulin. Cell.

[13] Ahluwalia N., Bergeron J.J., Wada I., Degen E., Williams D.B. (1992). The p88 molecular chaperone is identical to the endoplasmic reticulum membrane protein, calnexin. J. Biol. Chem..

[14] Degen E., Williams D.B. (1991). Participation of a novel 88-kd protein in the biogenesis of murine class i histocompatibility molecules. J. Cell Biol..

[15] Wada I., Rindress D., Cameron P.H., Ou W.J., Doherty J.J., Louvard D., Bell A.W., Dignard D., Thomas D.Y., Bergeron J.J. (1991). Ssr alpha and associated calnexin are major calcium binding proteins of the endoplasmic reticulum membrane. J. Biol. Chem..

[16] Pamer E., Cresswell P. (1998). Mechanisms of MHC class I-restricted antigen processing. Annu. Rev. Immunol..

[17] Rutkevich L.A., Williams D.B. (2011). Participation of lectin chaperones and thiol oxidoreductases in protein folding within the endoplasmic reticulum. Curr. Opin. Cell Biol..

[18] Kleizen B., Braakman I. (2004). Protein folding and quality control in the endoplasmic reticulum. Curr. Opin. Cell Biol..

[19] Caramelo J.J., Parodi A.J. (2008). Getting in and out from calnexin/calreticulin cycles. J. Biol. Chem..

[20] Oliver J.D., van der Wal F.J., Bulleid N.J., High S. (1997). Interaction of the thiol-dependent reductase erp57 with nascent glycoproteins. Science.

[21] Jessop C.E., Tavender T.J., Watkins R.H., Chambers J.E., Bulleid N.J. (2009). Substrate specificity of the oxidoreductase erp57 is determined primarily by its interaction with calnexin and calreticulin. J. Biol. Chem..

[22] Van Duyn Graham L., Sweetwyne M.T., Pallero M.A., Murphy-Ullrich J.E. (2010). Intracellular calreticulin regulates multiple steps in fibrillar collagen expression, trafficking, and processing into the extracellular matrix. J. Biol. Chem..

[23] Sugahara T., Koga T., Ueno-Shuto K., Shuto T., Watanabe E., Maekawa A., Kitamura K., Tomita K., Mizuno A., Sato T. (2009). Calreticulin positively regulates the expression and function of epithelial sodium channel. Exp. Cell Res..

[24] Ramos R.R., Swanson A.J., Bass J. (2007). Calreticulin and hsp90 stabilize the human insulin receptor and promote its mobility in the endoplasmic reticulum. Proc. Natl. Acad. Sci. USA.

[25] Okiyoneda T., Kono T., Niibori A., Harada K., Kusuhara H., Takada T., Shuto T., Suico M.A., Sugiyama Y., Kai H. (2006). Calreticulin facilitates the cell surface expression of abcg5/g8. Biochem. Biophys. Res. Commun..

[26] Arosa F.A., de Jesus O., Porto G., Carmo A.M., de Sousa M. (1999). Calreticulin is expressed on the cell surface of activated human peripheral blood t lymphocytes in association with major histocompatibility complex class i molecules. J. Biol. Chem..

[27] Sadasivan B., Lehner P.J., Ortmann B., Spies T., Cresswell P. (1996). Roles for calreticulin and a novel glycoprotein, tapasin, in the interaction of mhc class i molecules with tap. Immunity.

[28] White T.K., Zhu Q., Tanzer M.L. (1995). Cell surface calreticulin is a putative mannoside lectin which triggers mouse melanoma cell spreading. J. Biol. Chem..

[29] Orr A.W., Pedraza C.E., Pallero M.A., Elzie C.A., Goicoechea S., Strickland D.K., Murphy-Ullrich J.E. (2003). Low density lipoprotein receptor-related protein is a calreticulin coreceptor that signals focal adhesion disassembly. J. Cell Biol..

[30] Orr A.W., Elzie C.A., Kucik D.F., Murphy-Ullrich J.E. (2003). Thrombospondin signaling through the calreticulin/ldl receptor-related protein co-complex stimulates random and directed cell migration. J. Cell Sci..

[31] Stuart G.R., Lynch N.J., Day A.J., Schwaeble W.J., Sim R.B. (1997). The c1q and collectin binding site within c1q receptor (cell surface calreticulin). Immunopharmacology.

[32] Ogden C.A., deCathelineau A., Hoffmann P.R., Bratton D., Ghebrehiwet B., Fadok V.A., Henson P.M. (2001). C1q and mannose binding lectin engagement of cell surface calreticulin and cd91 initiates macropinocytosis and uptake of apoptotic cells. J. Exp. Med..

[33] Vandivier R.W., Ogden C.A., Fadok V.A., Hoffmann P.R., Brown K.K., Botto M., Walport M.J., Fisher J.H., Henson P.M., Greene K.E. (2002). Role of surfactant proteins a, d, and c1q in the clearance of apoptotic cells *in vivo* and *in vitro*: Calreticulin and cd91 as a common collectin receptor complex. J. Immunol..

[34] Malhotra R., Willis A.C., Jensenius J.C., Jackson J., Sim R.B. (1993). Structure and homology of human c1q receptor (collectin receptor). Immunology.

[35] Eggleton P., Lieu T.S., Zappi E.G., Sastry K., Coburn J., Zaner K.S., Sontheimer R.D., Capra J.D., Ghebrehiwet B., Tauber A.I. (1994). Calreticulin is released from activated neutrophils and binds to c1q and mannan-binding protein. Clin. Immunol. Immunopathol..

[36] Obeid M., Tesniere A., Ghiringhelli F., Fimia G.M., Apetoh L., Perfettini J.L., Castedo M., Mignot G., Panaretakis T., Casares N. (2007). Calreticulin exposure dictates the immunogenicity of cancer cell death. Nat. Med..

[37] Panaretakis T., Kepp O., Brockmeier U., Tesniere A., Bjorklund A.C., Chapman D.C., Durchschlag M., Joza N., Pierron G., van Endert P. (2009). Mechanisms of pre-apoptotic calreticulin exposure in immunogenic cell death. EMBO J..

[38] Tufi R., Panaretakis T., Bianchi K., Criollo A., Fazi B., Di Sano F., Tesniere A., Kepp O., Paterlini-Brechot P., Zitvogel L. (2008). Reduction of endoplasmic reticulum Ca^2+^ levels favors plasma membrane surface exposure of calreticulin. Cell Death Differ..

[39] Molinari M., Eriksson K.K., Calanca V., Galli C., Cresswell P., Michalak M., Helenius A. (2004). Contrasting functions of calreticulin and calnexin in glycoprotein folding and er quality control. Mol. Cell.

[40] Popescu C.I., Paduraru C., Dwek R.A., Petrescu S.M. (2005). Soluble tyrosinase is an endoplasmic reticulum (er)-associated degradation substrate retained in the er by calreticulin and bip/grp78 and not calnexin. J. Biol. Chem..

[41] Herrada G., Dulac C. (1997). A novel family of putative pheromone receptors in mammals with a topographically organized and sexually dimorphic distribution. Cell.

[42] Ryba N.J., Tirindelli R. (1997). A new multigene family of putative pheromone receptors. Neuron.

[43] Matsunami H., Buck L.B. (1997). A multigene family encoding a diverse array of putative pheromone receptors in mammals. Cell.

[44] Dey S., Matsunami H. (2011). Calreticulin chaperones regulate functional expression of vomeronasal type 2 pheromone receptors. Proc. Natl. Acad. Sci. USA.

[45] Martin V., Groenendyk J., Steiner S.S., Guo L., Dabrowska M., Parker J.M., Muller-Esterl W., Opas M., Michalak M. (2006). Identification by mutational analysis of amino acid residues essential in the chaperone function of calreticulin. J. Biol. Chem..

[46] Clouse S.D. (2002). Brassinosteroid signal transduction: Clarifying the pathway from ligand perception to gene expression. Mol. Cell.

[47] Jin H., Yan Z., Nam K.H., Li J. (2007). Allele-specific suppression of a defective brassinosteroid receptor reveals a physiological role of uggt in er quality control. Mol. Cell.

[48] Jin H., Hong Z., Su W., Li J. (2009). A plant-specific calreticulin is a key retention factor for a defective brassinosteroid receptor in the endoplasmic reticulum. Proc. Natl. Acad. Sci. USA.

[49] Hong Z., Jin H., Fitchette A.C., Xia Y., Monk A.M., Faye L., Li J. (2009). Mutations of an alpha1,6 mannosyltransferase inhibit endoplasmic reticulum-associated degradation of defective brassinosteroid receptors in arabidopsis. Plant Cell.

[50] Hong Z., Kajiura H., Su W., Jin H., Kimura A., Fujiyama K., Li J. (2012). Evolutionarily conserved glycan signal to degrade aberrant brassinosteroid receptors in arabidopsis. Proc. Natl. Acad. Sci. USA.

[51] Su W., Liu Y., Xia Y., Hong Z., Li J. (2011). Conserved endoplasmic reticulum-associated degradation system to eliminate mutated receptor-like kinases in arabidopsis. Proc. Natl. Acad. Sci. USA.

[52] Cheng S.H., Gregory R.J., Marshall J., Paul S., Souza D.W., White G.A., O'Riordan C.R., Smith A.E. (1990). Defective intracellular transport and processing of cftr is the molecular basis of most cystic fibrosis. Cell.

[53] Harada K., Okiyoneda T., Hashimoto Y., Oyokawa K., Nakamura K., Suico M.A., Shuto T., Kai H. (2007). Curcumin enhances cystic fibrosis transmembrane regulator expression by down-regulating calreticulin. Biochem. Biophys. Res. Commun..

[54] Karnabi E., Qu Y., Yue Y., Boutjdir M. (2013). Calreticulin negatively regulates the surface expression of cav1.3 l-type calcium channel. Biochem. Biophys. Res. Commun..

